# Dapagliflozin Mitigates Hypotension in Lipopolysaccharide-Induced Acute Inflammation Independent of Glycemia Level

**DOI:** 10.3390/pharmaceutics15061683

**Published:** 2023-06-08

**Authors:** Wael A. Alanazi, Turki Alharbi, Doaa M. El-Nagar, Abdullah M. Albogami, Mohammed Alswayyed

**Affiliations:** 1 Department of Pharmacology and Toxicology, College of Pharmacy, King Saud University, Riyadh 11451, Saudi Arabia; 2 Department of Zoology, College of Science, King Saud University, Riyadh 11451, Saudi Arabia; 3 Department of Pathology, College of Medicine, King Saud University, Riyadh 11451, Saudi Arabia

**Keywords:** dapagliflozin, lipopolysaccharide, hypotension, inflammation, diabetes, iNOS

## Abstract

Sodium-glucose cotransporter-2 (SGLT2) inhibitors have been suggested to have anti-inflammatory properties in diabetes. The goal of this study was to evaluate the role of the SGLT2 inhibitor dapagliflozin (DAPA) in the attenuation of lipopolysaccharide (LPS)-induced hypotension. Male Wistar albino rats were divided into normal and diabetic groups and received DAPA (1 mg/kg/day) for two weeks followed by a single dose of 10 mg/kg LPS. Blood pressure was recorded throughout the study and the circulatory levels of cytokines were assessed using a multiplex array, while the aortas were harvested for analysis. DAPA attenuated the vasodilation and hypotension caused by LPS. Mean arterial pressure (MAP) was preserved in the normal and diabetic DAPA-treated septic groups (MAP = 83.17 ± 5.27, 98.43 ± 5.57 mmHg) compared to the vehicle-treated septic groups (MAP = 65.60 ± 3.31, 68.21 ± 5.88 mmHg). Most of the cytokines induced by LPS were decreased in the DAPA-treated septic groups. In the aorta, the inducible nitric oxide synthase-derived nitric oxide had lower expression in the DAPA-treated rats. In contrast, the expression of α-smooth muscle actin, a marker of the vessel’s contractile state, was higher in the DAPA-treated rats in comparison with non-treated septic rats. These findings revealed that the protective role of DAPA against LPS-induced hypotension is likely to be glucose-lowering independent, as was observed in the non-diabetic septic group. Taken together, the results show that DAPA has a potential effect in the prevention of the hemodynamic disturbances of sepsis regardless of glycemia levels.

## 1. Introduction

Sepsis is a life-threatening condition characterized by extensive physiological abnormalities in multiple organs due to infection [1]. In the early stage of sepsis, the induction of inflammatory mediators causes endotoxemia, which frequently develops into septic shock, leading to necrosis of the kidneys, hemorrhages, hypotension, and myocardial dysfunction [2,3,4]. Lipopolysaccharide (LPS) is a fundamental structural component of the outer membrane of Gram-negative bacteria-induced sepsis resulting from severe inflammatory conditions linked to vascular dysfunction and hypotension [5,6]. It is a primary trigger factor in immunity characterized by cytokine-induced inducible nitric oxide synthase (iNOS) and activation of the immune response [7]. Sepsis caused by LPS affects several signaling pathways leading to heart failure (HF) by stimulating various cells to release pro-inflammatory mediators and other factors causing cell damage [7,8]. The upregulation of iNOS is the main component of sepsis-induced systemic inflammation and hypotension through a massive release of nitric oxide (NO) [5,9].

Patients with diabetes tend to have a greater susceptibility to infection due to a defect in the innate and adaptive immune system, and the severity of infection increases in uncontrolled glycemia [10]. The risk of sepsis incidence is two to six times higher in diabetic patients, representing the largest population of septic patients [10]. Experimental studies have shown that the clearance of sepsis is significantly reduced in type-1 diabetic mice, leading to higher induction of pro-inflammatory mediators [11]. Diabetic mice infected with Pseudomonas aeruginosa showed a direct relationship between hyperglycemia and the growth rate of the microorganisms in the liver, kidney, and spleen [11]. In vitro studies demonstrated that hyperglycemia impairs the role of innate immunity against pathogens in diabetic models, but the mechanism is still poorly understood [11]. In type-2 diabetic patients, chronic hyperglycemia disrupts the homeostasis of innate immunity, causing early organ dysfunction during septic conditions [12].

In diabetes management, growing interest has been shown in natural products that could be used to inhibit sodium-glucose cotransporter-2 (SGLT2). In the last decade, flavonoid phlorizin, a lead compound with SGLT2 inhibitory actions, which has been extracted from apple tree bark, has been discovered to have an SGLT2 inhibitory effect [13]. SGLT2 inhibitors are currently considered one of the novel classes of anti-diabetic drugs, which produce glycosuric and naturalistic effects by blocking renal reabsorption of glucose from the proximal convoluted tubules [14]. Thus, adding SGLT2 inhibitors to insulin enhances glycemic regulation without increasing the possibility of insulin-related hypoglycemic episodes [15]. Recently, SGLT2 inhibitors have been found to provide many benefits, including cardiac and renal protection and an anti-inflammatory effect, but the mechanism behind these benefits remains poorly understood and controversial [16]. 

Dapagliflozin (DAPA) is a potent and selective SGLT2 inhibitor [17]. In European countries, DAPA is the first oral therapy approved as an adjunctive treatment for type-1 diabetic patients with body mass index (BMI) ≥ 27 kg/m^2^ when optimal insulin therapy alone does not produce effective glycemic control [18,19]. Clinical trials have approved that DAPA reduces the risk of chronic kidney disease (CKD) and heart failure (HF), leading to a decline in deaths caused by renal and cardiovascular events in diabetic patients with CKD or HF with preserved ejection fraction (HFpEF) [20,21]. These beneficial effects have also been shown with another SGLT2 inhibitor, empagliflozin; the diabetic patients who received empagliflozin had better renal and cardiac outcomes in HF with reduced ejection fraction (HFrEF), HFpEF, and CKD [22,23,24]. Recent studies have shown a decisive influential role of DAPA in normalized inflammatory mediators leading to the prevention of apoptosis, atherosclerotic aortic lesions, and cardiovascular complications in diabetes [19]. In normal and diabetic people with progressive kidney failure, DAPA decreased the risk of kidney failure, mortality from coronary diseases, hospitalization for HF, and extended survival, regardless of the prevalence of concomitant cardiovascular diseases (CVD) [25]. In several clinical trials, there have been positive outcomes of the use of DAPA on cardiac functions, which might be related to the protection of the coronary endothelium parallel to the glycemia-related action [26]. 

Still, there is a lack of evidence about the protective role of DAPA against LPS-induced CVDs. This study explored the beneficial effects of DAPA in the prevention of blood sepsis complications involving hypotension. In-vivo studies were conducted to investigate the role of DAPA in the attenuation of cytokine-induced nitric oxide release and modulation of hypotension induced by LPS. In addition, this study was performed using wild-type and diabetic rat models to identify the protective role of DAPA against LPS is dependent or independent of glycemia levels. 

## 2. Materials and Methods

### 2.1. Animals

All animal experiments described in this study followed the National Institutes of Health (NIH) guidelines for the care and use of laboratory animals and were approved by the local institutional research ethics committee of King Saud University (KSU-SE-20-48). Forty-eight Wistar albino rats weighing 220–230 g were obtained from the Animal Care Center, College of Pharmacy, King Saud University (Riyadh, Saudi Arabia). The rats were fed with a standard chow pellet diet and had free access to water under controlled conditions (25 °C and a 12 h light/dark cycle).

### 2.2. Diabetes Induction

Twenty-four rats were treated with a single dose of streptozotocin (STZ) (Sigma-Aldrich, St. Louis, MO, USA) (60 mg/kg, i.p.) freshly dissolved in 0.1 mM sodium citrate buffer adjusted to pH 4.5 [27]. After injection, a 5% sucrose solution was provided for 48 h to avoid hypoglycemic shock. In addition, twenty-four rats were treated with a single dose of 0.1 mM sodium citrate buffer as a vehicle for the non-diabetic groups. Blood was obtained from the rat tail vein on the fifth day to measure fasting blood glucose (FBG) levels using a glucometer (ACCU-Chek Performa, Basel, Switzerland) to confirm each rat’s diabetic stage (250 mg/dL) [27].

### 2.3. Treatment and LPS-Induced Sepsis

**Figure 1:**
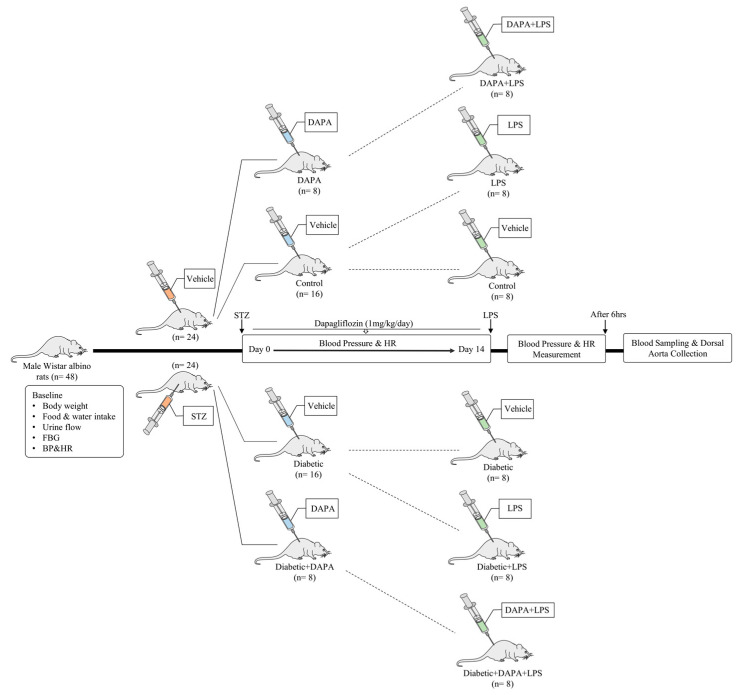
Schematic illustration of experimental protocols to study the effect of dapagliflozin on LPS-induced hypotension in normal and diabetic rat models. FBG, fasting blood glucose; BP, blood pressure; HR, heart rate; STZ, streptozotocin; DAPA, dapagliflozin; LPS, lipopolysaccharide.

The non-diabetic rats (n = 24) were divided randomly into three groups; the first group (n = 16) was treated with dimethyl sulfoxide (DMSO) < 1% as vehicle control for 2 weeks. On day 15, this group was divided into two groups: the vehicle group (n = 8) and the LPS group (n = 8). The vehicle group was treated with a single dose of saline, and the LPS group was treated with a single dose of lipopolysaccharide (LPS, 10 mg/kg, i.p.) (Sigma-Aldrich, St. Louis, MO, USA). The third group (DAPA + LPS) (n = 8) was treated daily with dapagliflozin (DAPA, 1 mg/kg, p.o.) (AK Scientific, Inc. CA, USA) for 2 weeks, followed by a single dose of LPS on day 15 (Figure 1).

The diabetic rats (n = 24) were divided into three groups; the diabetic group (n = 16) was treated with DMSO as vehicle control for 2 weeks. On day 15, this group was divided into two groups: the diabetic group (n = 8) and the diabetic + LPS group (n = 8). The diabetic group was treated with a single dose of saline, and the diabetic + LPS group was treated with a single dose of LPS (10 mg/kg, i.p.). The third group (diabetic + DAPA + LPS) (n = 8) was treated daily with DAPA (1 mg/kg, p.o.) for two weeks followed by a single dose of LPS on day 15 (Figure 1). The doses of DAPA and LPS and the routes of administration were selected following previous protocols [28,29].

FBG levels, food intake (FI), body weight (BW), and weight gain (WG) percentage were measured for all rats on days 0, 4, 8, 12, and 14. After six hours of LPS treatment, all rats were anesthetized by an intraperitoneal ketamine/xylazine mixture. Whole blood was immediately collected, and serum separation was done at 3000 *g* for 10 min (min) and kept at −80 °C until analysis. Dorsal aorta samples were washed with ice-cold phosphate buffer saline (PBS) and then fixed in a 4% formaldehyde solution for histopathology studies. Other aorta samples were snap-frozen in liquid nitrogen and stored at −80 °C until analysis using western blot analysis and biochemical assays.

### 2.4. Blood Pressure and Heart Rate Measurement

Systolic blood pressure (SBP), diastolic blood pressure (DBP), mean arterial pressure (MAP), and heart rate were measured in all rats after adaptation using a non-invasive tail-cuff CODA system (Kent Scientific, Torrington, CT, USA) every two days until day 12 as per protocol [30]. On day 15, blood pressure and heart rate were measured by the CODA system before and after three hours of LPS treatment.

### 2.5. Histopathology

The fixed aortas were embedded in paraffin and thin sections of 3–4 μm were prepared using a microtome. Then, paraffin-embedded sections were deparaffinized and stained with hematoxylin and eosin (H&E) [31]. The prepared slides were evaluated by a specialized histopathologist to identify any structural changes in the aortic tissues.

### 2.6. Cytokine Analysis

The collected serum samples were analyzed for measurement of cytokine/chemokine levels after LPS treatment in the normal and diabetic groups. This assay was performed to test 25 rat cytokine/chemokine levels including, eotaxin, fractalkine, IFNγ, IL-1α, IL-1β, IL-2, IL-4, IL-5, IL-6, IL-10, IL-12p70, IL-13, IL-17A, IL-18, IP-10, GRO/KC, TNFα, G-CSF, GM-CSF, MCP-1, Leptin, LIX, MIP-1α, MIP-2, VEGF-A. All cytokines were simultaneously measured in a single microwell by the Rat Cytokine/Chemokine Plex Discovery Assay^®^ Array (Eve Technologies, Calgary, AB, Canada).

### 2.7. Western Blotting

First, aortic segments (40 mg) were homogenized in radio-immunoprecipitation assay (RIPA) buffer (400 µL) mixed with 4 µL of protease inhibitor (Santa Cruz Biotechnology, Dallas, TX, USA). Protein lysates were incubated for 1 h on ice and vortexed every 15 min followed by centrifuging at 12,000 rpm for 15 min at 4 °C. Then, the supernatants of samples were collected and stored at −20 °C for further experiments. The protein concentration for each sample was quantified using the direct card method via Direct Detect^®^ Infrared Spectrometer (Merck Millipore, Burlington, MA, USA). Sample proteins were mixed with distilled water and 2× Laemmli sample buffer (Santa Cruz Biotechnology, Dallas, TX, USA) to prepare them for use in a western blot run. Next, samples were denatured by heating them at 50 °C for 5–10 min. Then, 30 µg of protein samples per lane were loaded on 12.5% SDS-polyacrylamide gel at 110 volts for 70–80 min. Then, proteins were transferred into nitrocellulose membranes (Santa Cruz Biotechnology, Dallas, TX, USA) using a semi-dry transfer instrument at 18 volts for 1 h (Bio-Rad, Hercules, CA, USA). The membrane was blocked with bovine serum albumin (BSA) in a washing buffer (TBST) for 1–2 h. The membrane was then incubated overnight on the shaker at 4 °C with rabbit polyclonal antibodies against iNOS (1:1000), α-smooth muscle actin (α-SMA) (1:1000), and β-actin (1:10,000) protein (ABclonal Technology, Woburn, MA, USA). The next day, the membranes were washed 3–5 times with TBST for 5 min. After that, the membranes were incubated with fresh HRP goat anti-rabbit IgG antibody (1:10,000) (Santa Cruz Biotechnology, Dallas, TX, USA) for 1–2 h. Then, the membranes were washed every 5 min with 3–5 times with TBST. Protein band expressions were visualized through the ChemiDoc MP imaging system (Bio-Rad, Hercules, CA, USA) using a western blotting luminol reagent (Santa Cruz Biotechnology, Dallas, TX, USA). The Supplemental Information details western blot quantification graphs, and provides full-length western blot images.

### 2.8. Nitric Oxide Assay

Aortic homogenates were deproteinated using zinc sulfate (ZnSO4) and sodium hydroxide (NaOH) for assessment of nitric oxide (NO) levels based on the enzymatic conversion of nitrate to nitrite by nitrate reductase using the Griess method via the QuantiChrom^TM^ Nitric Oxide Assay Kit (Bioassays Systems, Hayward, CA, USA). In accordance with the manufacturer’s instructions, the samples were run in a 96-wells plate and read at 540 nm.

### 2.9. Statistical Analysis

Data were expressed as means ± standard error of the mean (SEM) and analyzed by one-way or two-way analysis of variance (ANOVA) followed by Tukey’s multiple comparisons test when appropriate using GraphPad Prism 9 (San Diego, CA, USA). *P* values less than 0.05 were considered statistically significant.

## 3. Results

### 3.1. DAPA Regulates FBG Levels, Water/Food Intake, Weight Gain, and Urine Flow in Diabetic Rats

**Figure 2:**
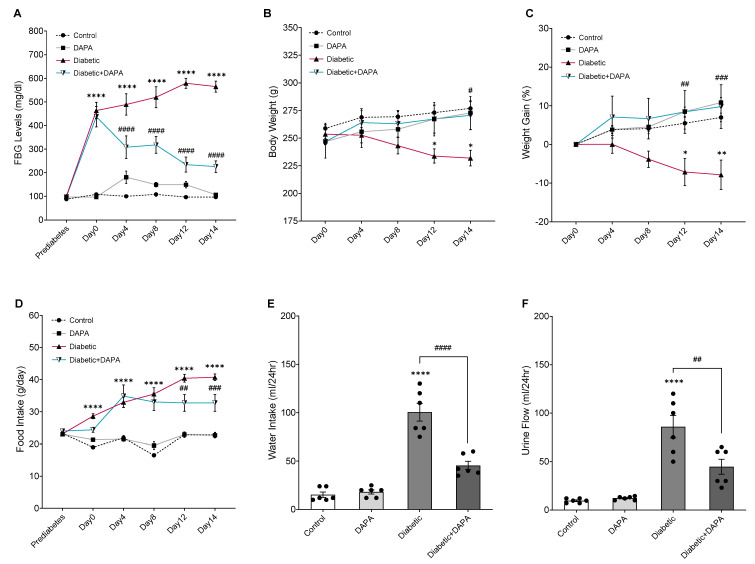
Effect of DAPA on FBG levels, body weight, food intake, water intake, and urination before LPS treatment. Normal and diabetic rats either daily received dapagliflozin (DAPA, 1 mg/kg, p.o.) or vehicle control. (**A**) Fasting blood glucose levels were measured before diabetes induction, and every four days using a glucometer. (**B**) Body weight measurement every four days. (**C**) The weight gain percentage was calculated every four days using the body weight measurement. (**D**) Food intake every four days. (**E**) Water intake. (**F**) Urine flow measurement was performed using a metabolic cage. Values are expressed as the mean ± SEM (n = 6−8). * *p* < 0.05, ** *p* < 0.01, **** *p* < 0.0001 (Control vs. Diabetic). ^#^ *p* < 0.05, ^##^ *p* < 0.01, ^###^ *p* < 0.001, ^####^ *p* < 0.0001 (Diabetic vs. Diabetic + DAPA).

FBG levels dramatically decreased during DAPA treatment in the diabetic + DAPA group from day 4 until the end of the study compared with day 0 in the same group and the untreated diabetic group (Figure 2A). DAPA treatment maintained the body weight of diabetic rats within the normal range due to improving the weight gain compared with the control and the diabetic group (Figure 2B,C). In addition, food/water intake and urine flow decreased during DAPA treatment in the diabetic + DAPA group compared to the diabetic group (Figure 2D–F), which might be related to the regulation of blood glucose levels in diabetes.

### 3.2. DAPA Regulates Blood Pressure in Diabetic Rats

**Figure 3:**
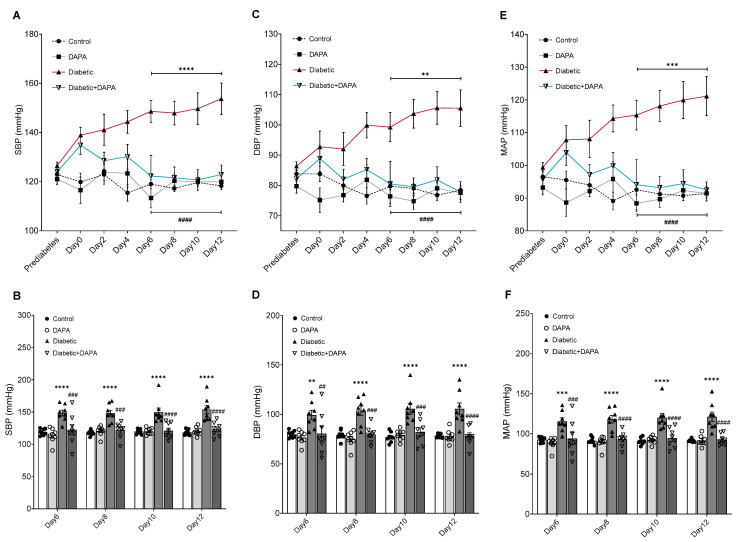
Effect of DAPA on blood pressure before LPS treatment. Normal and diabetic rats either received dapagliflozin (DAPA, 1 mg/kg, p.o.) or vehicle control daily. (**A**,**B**) Systolic blood pressure (SBP), (**C**,**D**) Diastolic blood pressure (DBP), and (**E**,**F**) Mean arterial pressure (MAP) were measured before diabetes induction, and every two days using a non-invasive tail-cuff CODA system. Values are expressed as the mean ± SEM (n = 8). ** *p* < 0.01, *** *p* ˂ 0.001, **** *p* ˂ 0.0001 (Control vs. Diabetic). ^##^ *p* < 0.01, ^###^ *p* < 0.001, ^####^ *p* < 0.0001 (Diabetic vs. Diabetic + DAPA).

During the two weeks of DAPA treatment, blood pressure was monitored every two days to evaluate the hemodynamic changes. The results showed an elevation in blood pressure parameters (SBP, DBP, and MAP) from day 0 until day 12 in comparison with the prediabetic condition in the diabetic group (Figure 3A–F). SBP, DBP, and MAP increased from 126.45 ± 1.46, 86.37 ± 1.46, and 99.41 ± 1.41 mmHg respectively before induction of diabetes to 138.92 ± 3.17, 92.72 ± 5.24, and 107.77 ± 4.35 mmHg on day 0, and they continuously increased to reach 153.70 ± 6.40, 105.48 ± 6.04, and 121.15 ± 5.96 mmHg on day 12 in the diabetic group (Figure 3A). In DAPA treatment, SBP, DBP, and MAP decreased from 134.83 ± 3.75, 88.85 ± 4.19, and 103.87 ± 3.93 mmHg on day 0 until they reached 122.90 ± 3.83, 77.80 ± 3.42, and 92.51 ± 2.43 mmHg on day 12 in the diabetic + DAPA group (Figure 3A–F). DAPA treatment resulted in a significant reduction in SBP, DBP, and MAP such that they reached normal levels on days 6, 8, 10, and 12 in the diabetic + DAPA group compared to the control and diabetic groups (Figure 3B,D,F). These results suggest that DAPA as a monotherapy could prevent the disturbances in the hemodynamic function caused by diabetes.

### 3.3. DAPA Attenuates LPS-Induced Vasodilation and Hypotension

**Figure 4:**
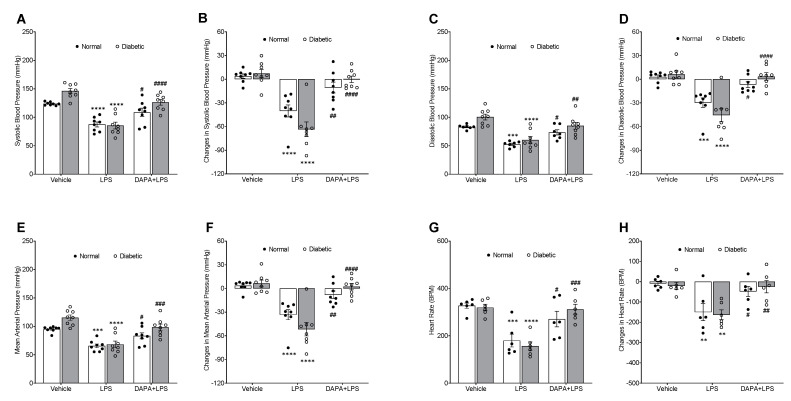
Effect of DAPA on LPS-induced hypotension. Normal and diabetic rats either daily received dapagliflozin (DAPA, 1 mg/kg, p.o.) or vehicle control, and on day 14 were injected with lipopolysaccharide (LPS, 10 mg/kg, i.p.). After three hours of LPS injection, (**A**) Systolic blood pressure (SBP), (**C**) Diastolic blood pressure (DBP), (**E**) Mean arterial pressure (MAP), and (**G**) Heart rate (HR) were measured using a non-invasive tail-cuff CODA system. The changes in SBP, DBP, MAP (**B**,**D**,**F**), and heart rate (**H**) were calculated by taking the differences between the values before and after sepsis. Values are expressed as the mean ± SEM (n = 6−8). ** *p* < 0.01, *** *p* ˂ 0.001, **** *p* ˂ 0.0001 (Vehicle vs. LPS). ^#^ *p* < 0.05, ^##^ *p* < 0.01, ^###^ *p* < 0.001, ^####^ *p* < 0.0001 (LPS vs. DAPA + LPS).

On day 15, blood pressure was recorded before and after three hours of LPS administration to assess the changes associated with blood sepsis in the normal and diabetic groups with or without DAPA. The changes in the blood pressure parameters and heart rate were calculated by taking the differences between the collected data before and after sepsis. After LPS-induced sepsis, blood pressure was significantly decreased, leading to hypotension in both the normal and diabetic groups (Figure 4A–F). In contrast, DAPA resisted the hemodynamic changes induced by sepsis and showed higher SBP, DBP, and MAP in the normal and diabetic groups compared with the non-treated LPS group (Figure 4A–F). In the normal rat model and after LPS treatment, SBP, DBP, and MAP were reduced significantly from 123.73 ± 1.02, 81.98 ± 1.66, and 94.96 ± 1.73 mmHg respectively in the vehicle group to 87.31 ± 4.36, 55.18 ± 3.04, and 65.60 ± 3.31 mmHg in the LPS group (Figure 4A,C,E). In the same model, DAPA prevented the reduction of blood pressure caused by sepsis and showed significantly higher levels of SBP (108.71 ± 7.12 mmHg), DBP (70.92 ± 4.60 mmHg), and MAP (83.17 ± 5.27 mmHg) compared to the LPS group (Figure 4A,C,E). 

In the diabetic groups, blood sepsis caused a significant reduction in SBP, DBP, and MAP from 146.12 ± 4.39, 100.38 ± 5.02, and 115.26 ± 4.71 mmHg respectively in the vehicle group to 85.34 ± 6.06, 60.14 ± 5.84, and 68.21 ± 5.88 mmHg in the LPS group (Figure 4A,C,E). In the diabetic DAPA + LPS group, DAPA regulated LPS-induced hypotension and showed higher values of SBP (126.11 ± 4.94 mmHg), DBP (85.06 ± 6.06 mmHg), and MAP (98.43 ± 5.57 mmHg) (Figure 4A,C,E). Moreover, heart rate was significantly decreased after LPS treatment, causing bradycardia in both the normal and diabetic groups compared to the vehicle groups (Figure 4G,H). In contrast, DAPA attenuated sepsis-induced bradycardia and decreased the difference in the heart rate before and after LPS injection in the DAPA + LPS groups (Figure 4G,H).

### 3.4. DAPA Prevents LPS-Induced Cytokines Production

**Figure 5:**
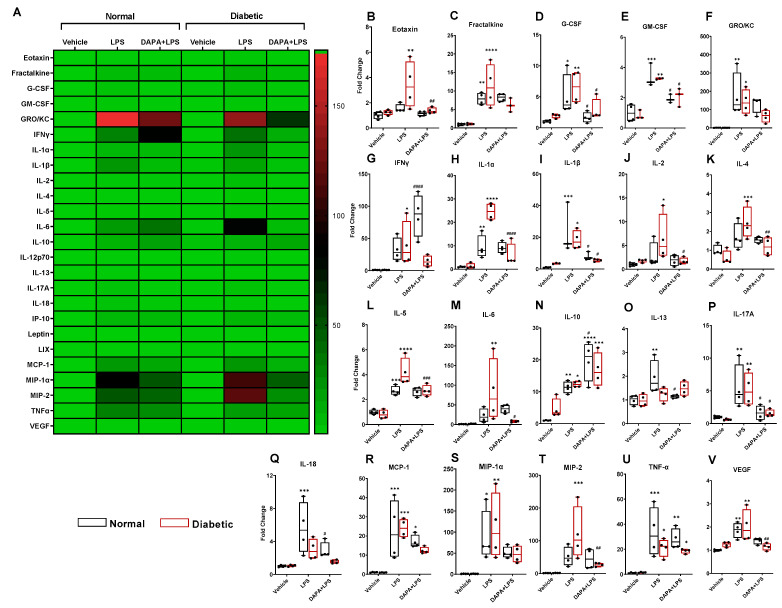
Effect of DAPA on cytokines production. Normal and diabetic rats either received dapagliflozin (DAPA, 1 mg/kg, p.o.) or vehicle control daily, and on day 14 were injected with lipopolysaccharide (LPS, 10 mg/kg, i.p.). (**A**) Heat map of the fold changes of cytokines/chemokines to the control in the normal and diabetic rats. (**B**–**V**) Bar graphs of the fold changes of cytokines/chemokines to the control in the normal and diabetic rats. Values are expressed as the mean ± SEM (n = 6–8). * *p* < 0.05, ** *p* < 0.01, *** *p* ˂ 0.001, **** *p* ˂ 0.0001 (compared to vehicle). ^#^ *p* < 0.05, ^##^ *p* < 0.01, ^###^ *p* < 0.001, ^####^ *p* < 0.0001 (compared to LPS).

Cytokines, including, eotaxin, fractalkine, IFNγ, IL-1α, IL-1β, IL-2, IL-4, IL-5, IL-6, IL-10, IL-12p70, IL-13, IL-17A, IL-18, IP-10, GRO/KC, TNFα, G-CSF, GM-CSF, MCP-1, Leptin, LIX, MIP-1α, MIP-2, and VEGF were assessed, and most of them were elevated after sepsis induction in both the normal and diabetic rats (Figure 5A–V). Fractalkine, G-CSF, GM-CSF, GRO/KC, IL-1α, IL-1β, IL-5, IL-10, IL-17A, MCP-1, MIP-1α, TNFα, and VEGF levels were significantly increased in the normal and diabetic septic rats (Figure 5C–F,H,I,L,N,P,R,S,U,V). In contrast, the septic groups treated with DAPA showed no significant changes in eotaxin, G-CSF, GM-CSF, IL-1β, IL-2, IL-4, IL-6, IL-13, IL-17A, IL-18, MIP-1α, MIP-2, and VEGF levels in comparison with the non-septic groups (Figure 5B,D,I,J,K,M,O–Q,S,T,V). In addition, DAPA significantly attenuated the induction of G-CSF, GM-CSF, IL-1β, and IL-17A in the normal and diabetic septic rats compared to the non-treated septic groups (Figure 5D,E,I,P). The results indicate that DAPA significantly counteracted IL-10, IL-13, and IL-18 release in the septic group with normal glycemia compared to the vehicle-treated septic group with normal glycemia (Figure 5N,O,Q), while DAPA significantly decreased eotaxin, fractalkine, IL-1α, IL-2, IL-4, IL-5, IL-6, and MIP-2 levels in the septic group with diabetes (Figure 5H,J–M,T).

### 3.5. DAPA Regulates Histological Changes, Nitric Oxide Production and Expression of iNOS and α-SMA in Aortas of Septic Rats

**Figure 6:**
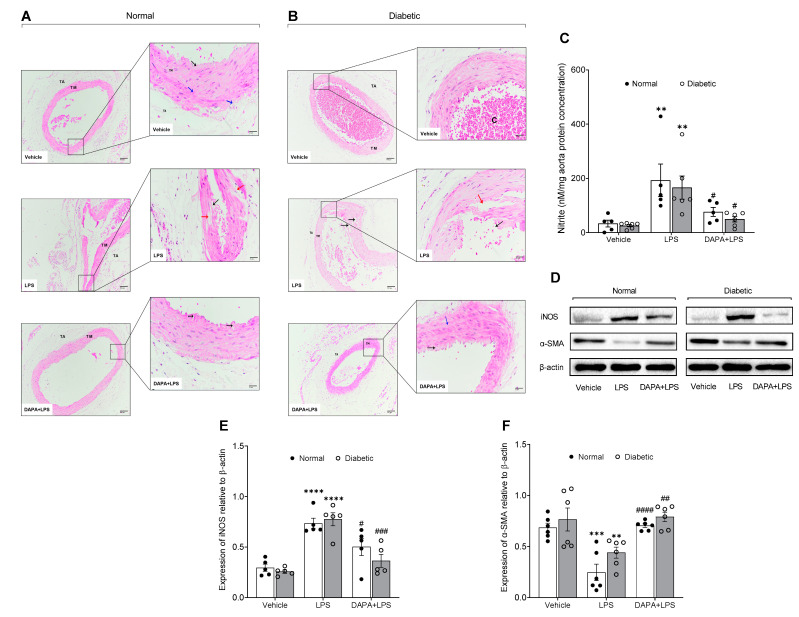
Effect of DAPA on aorta histology, nitric oxide production and protein expression of iNOS and α-SMA in sepsis-induced vasodilation. Normal and diabetic rats either daily received dapagliflozin (DAPA, 1 mg/kg, p.o.) or vehicle control, and on day 14 were injected with lipopolysaccharide (LPS, 10 mg/kg, i.p.). (**A**,**B**) Photomicrographs of the normal and diabetic rat dorsal aorta. (**A**) Vehicle aorta in the normal rat showing a normal structure with abundant tunica intima and thick media layer. (**A**) Aorta of rats treated with LPS showing wide dilatation, severe intima destruction, and muscle degeneration. (**A**) Aorta of rats treated with DAPA plus LPS showing less dilatation and degeneration of the intima layer. (**B**) Aorta of diabetic rats displaying congestion with blood cells. (**B**) Aorta of diabetic rats treated with LPS viewing wide dilatation and severe intima and media degeneration. (**B**) Aorta of diabetic rats treated with DAPA plus LPS indicating less dilatation without congestion and abundant intima layer. (TM) tunica media, (TA) tunica adventitia, (black arrows) tunica intima, (blue arrow) elastic fibers, and (red arrow) muscle degeneration. (HE-100-400X). (**C**) Nitrite levels in the aorta. (**D**) Representative data from western blot analysis of iNOS, α-SMA, and β-actin protein expression in the aorta. (**E**) Quantification of iNOS protein levels. (**F**) Quantification of α-SMA protein levels. ** *p* < 0.01, *** *p* ˂ 0.001, **** *p* ˂ 0.0001 (Vehicle vs. LPS). ^#^ *p* < 0.05, ^##^ *p* < 0.01, ^###^ *p* < 0.001, ^####^ *p* < 0.0001 (LPS vs. DAPA + LPS).

In the histological study, rat dorsal aortas showed normal dilatation in the normal group treated with a vehicle (Figure 6A). Aortal structure with internal tunica intima consists mainly of endothelial cells facing the lumen beneath with a small layer of connective tissue followed by elastic lamina, tunica media that consists of a thick smooth muscle layer alternating with elastic fibers and tunica adventitia, which consists of loose connective tissue interspersed with elastic fibers (Figure 6A). Dorsal aortas of normal rats treated with LPS revealed marked pathological changes manifested by wide dilatation of the aorta with obvious deformation that led to the destruction of the tunica intima in addition to muscle degeneration (Figure 6A). In contrast, the aortas of normal rats treated with LPS and DAPA showed noticeable improvement represented by a healthy appearance and a good-looking muscle layer with the exception of some degeneration of the tunica intima (Figure 6A). The aortas of diabetic rats displayed congestion with erythrocytes and much thickening of the media muscle layer, which could lead to hypertension (Figure 6B). Furthermore, treatment of diabetic rats with LPS resulted in wide dilatation of the aortas with media degeneration, severe degeneration of the intima layer, and wide muscle degeneration areas (Figure 6B). Moreover, the aortas of diabetic rats treated with LPS and DAPA exhibited great improvement characterized by approximately normal dilatation without congestion and healthy intima and media structures (Figure 6B).

On the molecular level, NO levels, iNOS, and α-SMA expression in the aorta were assessed to identify the mechanism underlying DAPA’s prevention of LPS-induced vasodilation. The aortic level of NO was assessed by measuring nitrite levels, which revealed a significant increase in nitrite levels caused by LPS (Figure 6B). In contrast, DAPA decreased nitrite levels in the normal and diabetic groups in the DAPA + LPS-treated group (Figure 6C). In addition, protein expression of iNOS in the aorta was increased by LPS, while DAPA modulated this effect in septic rats in normal and diabetic conditions (Figure 6D,E). In contrast, α-SMA protein expression was significantly suppressed in the normal and diabetic septic rats without treatment. Pre-treatment with DAPA reduced the downregulation of α-SMA protein expression induced by LPS (Figure 6D,F).

## 4. Discussion

The current study is the first to demonstrate that an SGLT2 inhibitor, namely dapagliflozin, attenuates hypotension caused by LPS-induced acute inflammation. DAPA prevented the related consequences of sepsis in the aortas of septic rats through regulation of iNOS and α-SMA expression independent of blood glucose levels. The main findings of this study are as follows. First, two weeks of DAPA treatment attenuated the systemic hemodynamic and metabolic disturbances associated with STZ-induced type-1 diabetes, including blood pressure, body weight, weight gain, and food/water intake. Second, pre-treatment with DAPA prevented sepsis-induced hypotension and bradycardia in normal and diabetic rats. Third, DAPA modulated systemic inflammation via regulation of the inflammatory mediators, including G-CSF, GM-CSF, IL-1β, and IL-17A in the LPS-treated rats with and without diabetes. Fourth, the histological studies showed that DAPA reduced the dilatation and deformation of the aortas of the septic rats. Fifth, DAPA decreased iNOS-derived NO, leading to the restoration of the protein expression of α-SMA to normal levels. Interestingly, the findings revealed that the anti-inflammatory effects of DAPA against LPS-induced hypotension are likely to be glucose-lowering independent, as was observed in the non-diabetic septic rat model. 

A large body of evidence has revealed that DAPA treatment provides metabolic and hemodynamic stability in both the short and long term for type-2 diabetes [32,33]. These findings are consistent with the current results, which demonstrate that DAPA attenuates disturbances in blood pressure, FBG levels, weight gain percentage, and food/water intake in STZ-induced type-1 diabetes. Thus, this study found that DAPA also has a beneficial effect on the modulation of hypertension and the related consequences of type-1 diabetes. Previous studies have found that DAPA treatment regulates blood pressure in both acute and chronic situations through a decrease in plasma volume, sympathetic nervous system activity, and vasoconstrictors and an increase of vasodilators, including atrial natriuretic peptide and cyclic guanosine monophosphate in type-2 diabetic subjects [32,33]. Recent studies have found that SGLT2 inhibition attenuates ischemia-reperfusion injury, leading to improved cardiac function via increasing cardiac salvage and preserving cardiac output, which might regulate the hemodynamic function and prevent hypotension caused by reperfusion injury [34]. SGLT2 inhibition also showed beneficial effects in enhancing energetics that were impaired during blood sepsis, as shown previously [35,36].

Bacterial LPS administration has been widely used to induce blood sepsis causing hypotension and bradycardia as shown in this study and several previous studies [37,38]. The hemodynamic alteration in septic shock has been correlated with the induction of inflammatory mediators, which are induced through the activation of several signaling pathways including toll-like receptor-4 (TLR4), mitogen-activated protein kinase (MAPK), nuclear factor kappa B (NF-κB), and activator protein-1 (AP-1) [38,39,40]. In the present study, array-based multiplexed screening revealed that DAPA notably decreased cytokine levels in the serum of normal and diabetic septic rat models. These results show that DAPA has a potential effect against LPS-induced cytokines regardless of glycemia levels. Similarly, a recent study found that DAPA alleviated LPS-induced acute kidney injury through attenuation of cytokine production and oxidative stress in a septic mice model [41]. Other recent evidence examined the link between SGLT2 inhibition and inflammation and found that DAPA exerts its anti-inflammatory effect via the downregulation of TLR4 and inhibition of NF-κB in colistin-induced nephrotoxicity [42]. Similar findings were obtained in human macrophages and endothelial cells; treatment with LPS and DAPA resulted in downregulation of the TLR4-induced NF-κB signaling pathway [43]. In addition, it has been found that SGLT2 inhibition exerts its anti-inflammatory effect by reducing the ratio of the proinflammatory M1 phenotype of macrophage to its anti-inflammatory M2 phenotype [44]. Still, the mechanisms underlying the anti-inflammatory roles of DAPA are controversial and not fully understood. Future studies should be conducted to find the association between the SGLT2 inhibition and the cellular mechanisms implicated in the induction of cytokines including TLR4, MAPK, and NF-κB. In addition, these interactions should be examined in euglycemic and hyperglycemic conditions to study the anti-inflammatory actions of SGLT2 inhibitors and determine whether they are dependent or independent of glycemia.

The current results show that the consequences of acute inflammation in the dilated aorta have been indicated via observation of severe dilatation with high expressed iNOS and increased NO bioavailability in the aortic tissues. Aortic dilatation was correlated to the downregulation of α-SMA, which is well-known as a marker of the vessel’s contractile state [45]. These findings are consistent with previous studies that showed that LPS hypotension mediated by induced iNOS causes overproduction of NO and decreases vasocontractile response in the blood vessels [46,47]. Studies have approved that knockout of iNOS reduces the NO formation in the LPS-induced hypotension compared to the wild-type mice model [46]. Thus, it has been revealed that NO release causing hypotension is solely dependent on the iNOS upregulation in septic animal models [46,47]. In addition, a mice model with tetrahydrobiopterin deficiency, the essential cofactor in NOS-derived NO, showed depletion of LPS-induced vascular dysfunction and hypotension, which also proved the critical role of this mechanism in causing hypotension by LPS [47]. Interestingly, the current results revealed that DAPA treatment inhibits LPS-induced vascular iNOS expression, leading to a decrease in NO production and a return to the expression of α-SMA in the aorta, which prevents the hypotensive effect of LPS in both normal and diabetic rat models. This study represents for the first time the potential role of DAPA in the prevention of hypotension caused by LPS through the attenuation of iNOS-derived NO independent of glycemia levels. Further studies should be conducted to investigate the off-target effect of DAPA in the downregulation of the Na+/H+ exchanger-1 (NHE1) during endotoxemia. NHE1 inhibition by DAPA mitigates cardiovascular injuries independent of glycemia as reported recently [48,49]. 

In summary, the current findings indicate that DAPA has a pivotal role in the attenuation of LPS-induced hypotension regardless of glycemia levels. In this study performed on non-diabetic and diabetic rat models with sepsis, DAPA prevented the induction of cytokines and iNOS in both normal and diabetic rat models. In addition, DAPA attenuated iNOS-induced NO in the aorta, improving its vascular integrity by maintaining α-SMA levels. These findings reflected on the histological results and blood pressure parameters; DAPA counteracted the vasodilator and hypotensive effects of LPS and maintained these parameters within normal values. Therefore, DAPA might provide a beneficial effect against sepsis-caused hypotension in non-diabetic and diabetic patients. The limitations of this study are centered on the insufficient results of the endothelial function needed to assess the response of the endothelial cells in the aorta to the stimulants in DAPA and/or LPS treatment. In addition, clinical experiments are required to confirm the beneficial effects of DAPA against sepsis-induced hypotension. Moreover, mechanistic studies are needed to investigate the crosstalk between NHE1, TLR4, MAPK, and NF-κB pathways and iNOS in DAPA and/or LPS treatment. 

In conclusion, the current study has revealed that DAPA has a potential protective role against the induction of hypotension by sepsis independent of glycemia level, which should be confirmed by future clinical investigations.

## Data Availability

Data is contained within the article.

## Supplementary Materials

The following supporting information can be downloaded at: https://www.mdpi.com/article/10.3390/pharmaceutics15061683/s1, Table S1: Quantitative western blot results; Figures: Full-length western blot images.
