# Supplementary material for: Dapagliflozin Mitigates Hypotension in Lipopolysaccharide-Induced Acute Inflammation Independent of Glycemia Level
Source: Pharmaceutics. 2023 Jun 8;15(6):1683. doi: 10.3390/pharmaceutics15061683 (PMC10301321; doi:10.3390/pharmaceutics15061683)

Supporting Information File to:

Dapagliflozin Mitigates Hypotension in Lipopolysaccharide-Induced Acute Inflammation Independent of Glycemia Level

Supplementary Table S1 contains supplementary raw data to Figure 6D, E.

Expression of iNOS relative to β-actin and respective *p*-value

	**Expression of iNOS relative to β-actin**									
	**Normal**					**Diabetic**				
Groups	N1	N2	N3	N4	N5	N1	N2	N3	N4	N5
Vehicle	0.347646	0.218546	0.404715	0.279796	0.229285	0.265847	0.206832	0.240439	0.262731	0.312916
LPS	0.673408	0.938612	0.662284	0.720089	0.67602	0.803036	0.818622	0.531084	0.812203	0.914793
DAPA+LPS	0.669541	0.182536	0.486178	0.565453	0.606609	0.279599	0.268577	0.468614	0.239473	0.565016
P-value	Normal<br>Vehicle vs. LPS *P* <0.0001<br>Vehicle vs. DAPA+LPS *P*= 0.0463<br>LPS vs. DAPA+LPS *P*= 0.0230					Diabetic<br>Vehicle vs. LPS *P* <0.0001<br>Vehicle vs. DAPA+LPS *P*= 0.4039<br>LPS vs. DAPA+LPS *P*= 0.0001				

Supplementary Table S1 contains supplementary raw data to Figure 6D, F.

Expression of α-SMA relative to β-actin and respective *p*-value

	**Expression of α-SMA relative to β-actin**											
	**Normal**						**Diabetic**					
Groups	N1	N2	N3	N4	N5	N6	N1	N2	N3	N4	N5	N6
Vehicle	0.55274	0.643387	0.703181	0.606501	0.762112	0.843006	0.543663	0.499377	1.04536	0.932221	0.506979	1.065777
LPS	0.118418	0.120464	0.438067	0.072467	0.548548	0.186959	0.337255	0.447386	0.539472	0.556805	0.226145	0.534864
DAPA+ LPS	0.655254	0.707362	0.767793	0.65157	0.719375	0.72506	0.660369	0.649072	0.888854	0.792994	0.865565	0.89143
P-value	Normal<br>Vehicle vs. LPS *P* = 0.0002<br>Vehicle vs. DAPA+LPS *P* = 0.9772<br>LPS vs. DAPA+LPS *P* < 0.0001						Diabetic<br>Vehicle vs. LPS *P* = 0.0045<br>Vehicle vs. DAPA+LPS *P* = 0.9593<br>LPS vs. DAPA+LPS *P* = 0.0022					

Uncropped blots shown in Figure 6

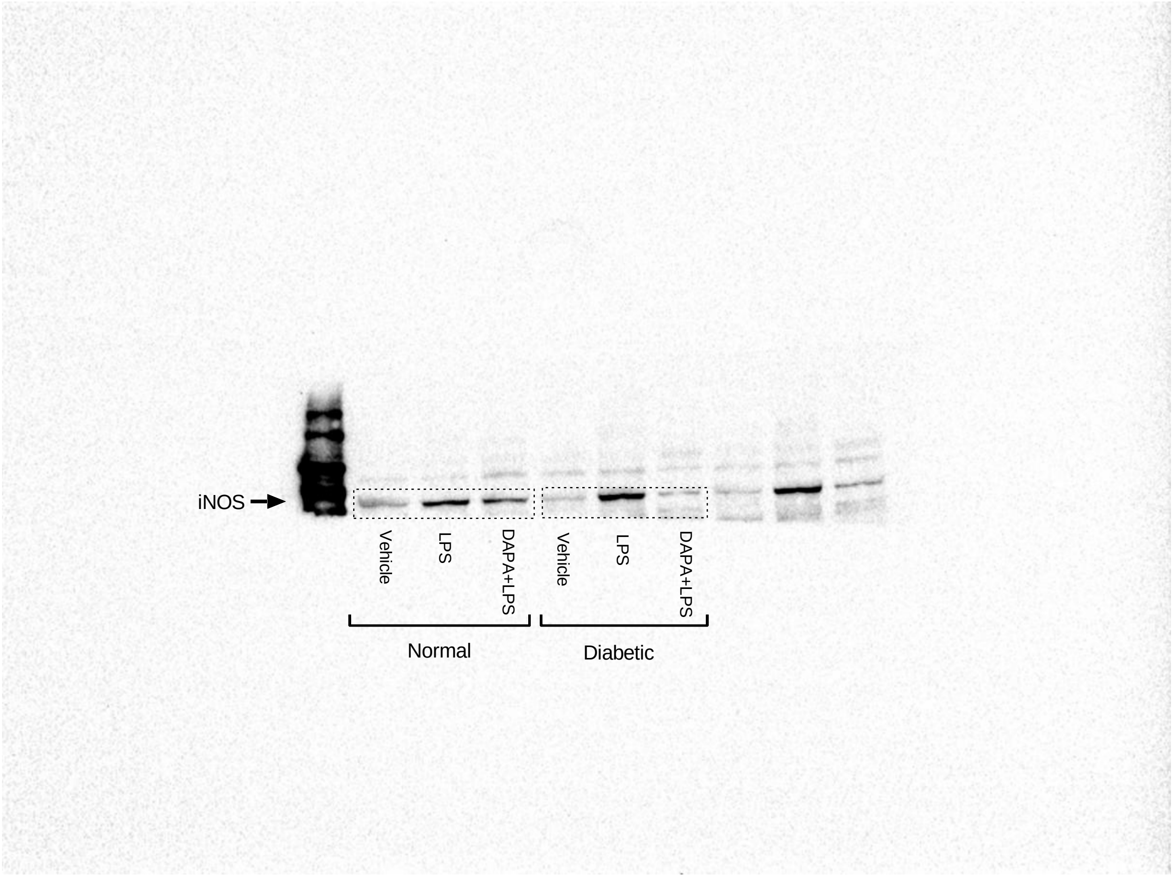

Uncropped blots shown in Figure 6

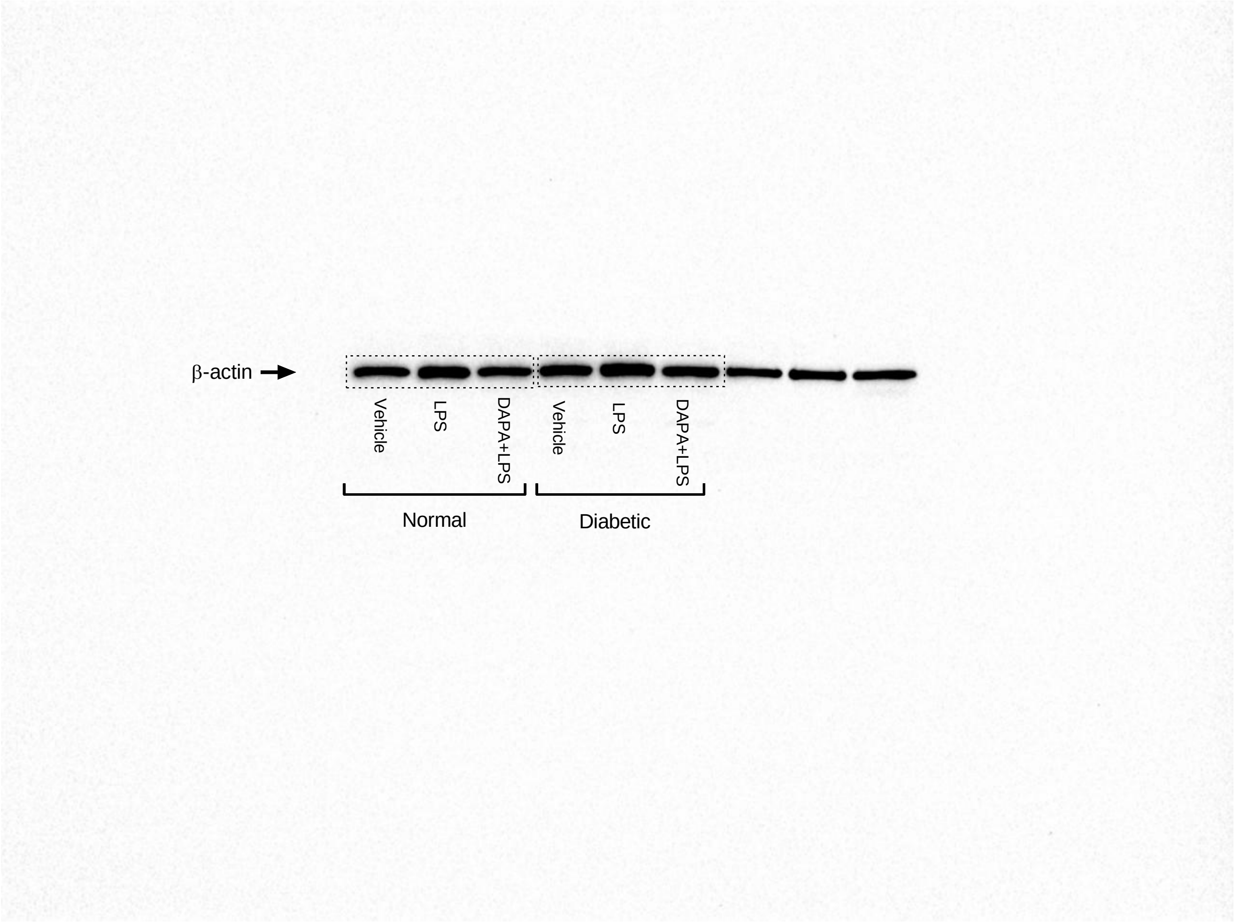

Uncropped blots shown in Figure 6

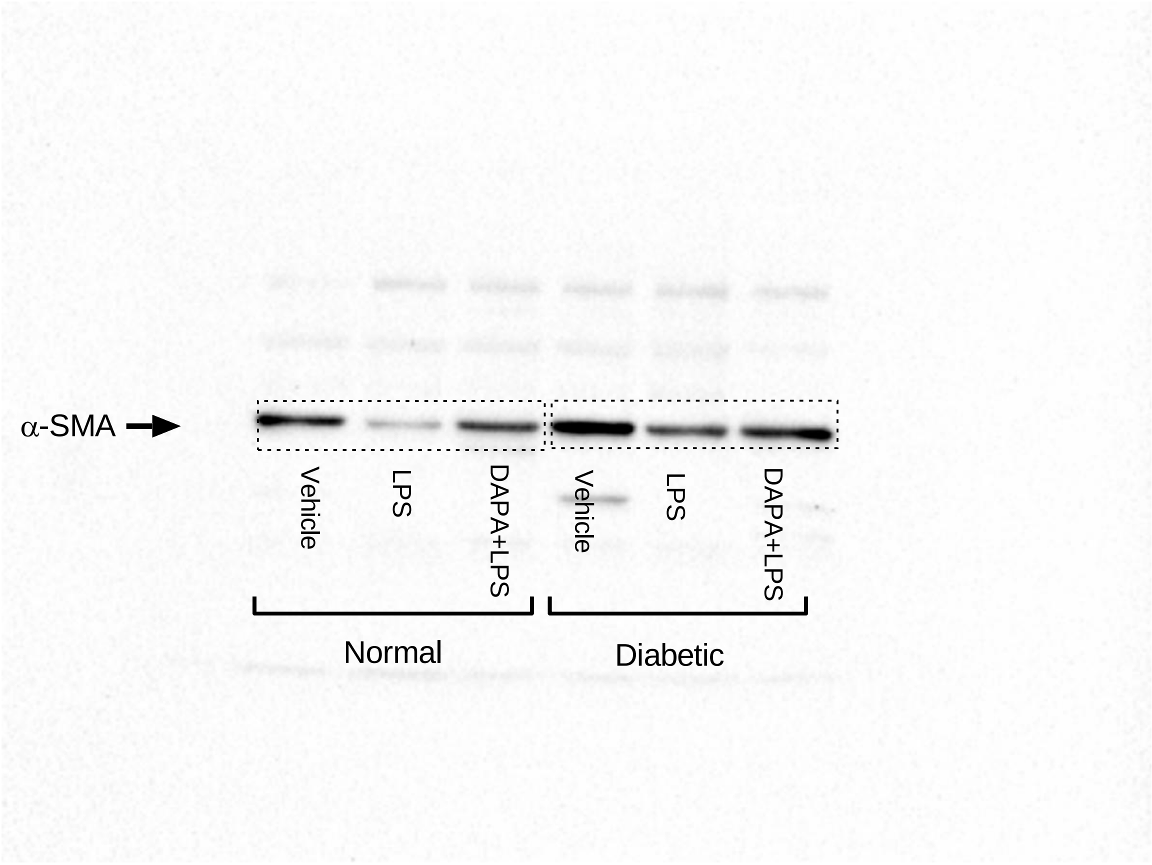

Uncropped blots shown in Figure 6

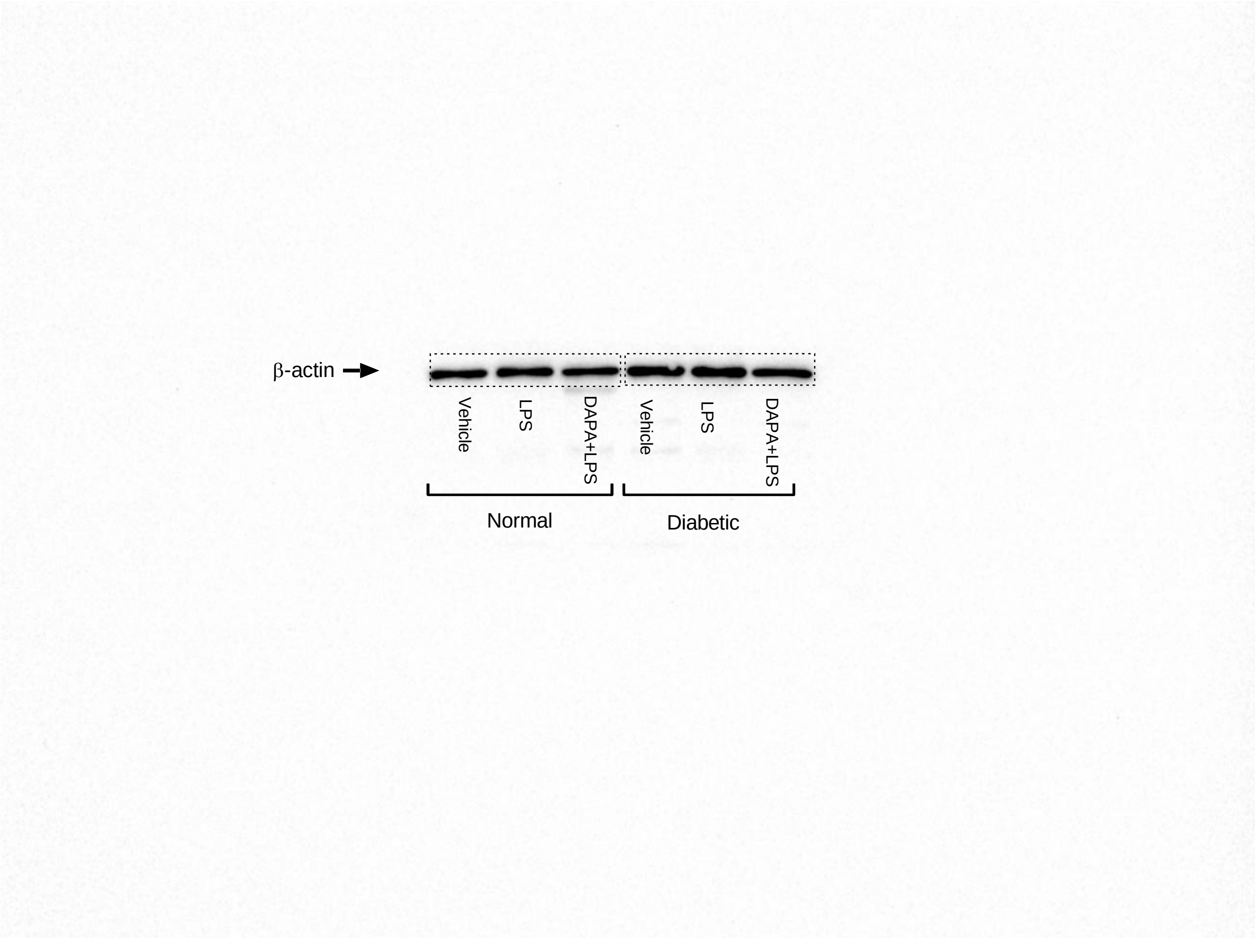

Supplement: Supplementary file 1 [file pharmaceutics-15-01683-s001.zip › pharmaceutics-2349383-supplementary.pdf]
